# Severe flea-borne typhus complicated by hemophagocytic lymphohistiocytosis: A case report and review of literature

**DOI:** 10.1016/j.idcr.2024.e01955

**Published:** 2024-04-15

**Authors:** Rolando A. Zamora Gonzalez, Mark S. Mayo, Arthur C. Jeng

**Affiliations:** a Infectious Diseases, University of California Los Angeles, Los Angeles, USA; b Department of Medicine, University of Washington, Seattle, USA; c Infectious Diseases, Olive View-UCLA Medical Center, Los Angeles, USA

**Keywords:** Flea-borne typhus, Hemophagocytic lymphohistiocytosis

## Abstract

Flea-borne typhus (FBT), also known as murine typhus, is a zoonotic infection caused by *R. typhi* with world-wide distribution. In the United States, the infection is uncommon but remains endemic in some areas, including Los Angeles County. It typically manifests as a benign acute febrile illness but can be complicated in a minority of cases. Associated hemophagocytic lymphohistiocytosis (HLH) has been described in a limited number of cases. Here, we present a case of a patient with FBT complicated by HLH treated empirically with doxycycline with subsequent resolution of HLH. Also included is a review of the literature of other published cases.

## Background

Flea-borne typhus, also known as murine typhus or endemic typhus, is a zoonosis that occurs in tropical and subtropical coastline regions in much of the world caused by the intracellular bacteria *Rickettsia typhi.* The bacteria is transmitted to humans by flea vectors from various hosts, traditionally rats but also cats and opossums [1]. The clinical presentation is non-specific, usually manifesting as an acute febrile illness associated with headache, myalgia, nausea, vomiting, and rash, with laboratory tests showing leukopenia, thrombocytopenia, mild to moderate transaminase elevation, hypoalbuminemia, and electrolyte abnormalities including hyponatremia and hypocalcemia [2]. In the United States, the disease reached peak incidence in the mid 1940 s with more than 5000 cases/year, with use of rodenticides resulting in a subsequent dramatic decline. Since then, the disease has remained endemic in parts of the United States, particularly southern Texas, southern California (predominantly in Los Angeles), and Hawaii. In recent years, Los Angeles County has experienced a resurgence in cases [3]. Here, we report a case of a patient with flea-borne typhus presenting with hemophagocytic lymphohistiocytosis (HLH) syndrome, a very rare and potentially fatal complication.

## Case presentation

A 32-year-old male with no significant past medical history presented to the emergency department of a university affiliated-county hospital in Los Angeles for evaluation of fever. The patient reported one week of nightly fever, chills, and drenching night sweats. He also reported feeling malaise, fatigue, myalgias, arthralgias, and headache. He had no recent sick contacts but did have a protected sexual encounter with a female 4 days prior to symptom onset. On review of systems, he denied odynophagia, respiratory, neurological, gastrointestinal, or genitourinary symptoms. He noticed a rash in his neck area that developed 2 weeks prior. Of note, he reported a similar rash 3 years prior that self-resolved after a few weeks.

On social history the patient was born in Hidalgo, Mexico and moved to the United States 6 years prior to presentation. He traveled the US for a year, including to Texas, Florida, Tennessee, Missouri, Montana, before settling in Sylmar, California where he presently resided. The patient lived in an apartment with his father, stepmother, and stepbrother. He reports working for a recycling company, receiving, and distributing materials/trash. As part of his job, he frequently found animals in the trash, both dead and alive, including dogs, cats, rats, squirrels, and opossums. He reported no known arthropod (including fleas and ticks) or animal bites. He denied consumption of unpasteurized dairy products. He reported being sexually active with female partners, with no history of sexually transmitted infections. He reported regular alcohol use, up to 15 beers per day for the prior 2 months, smoked half a pack per day of cigarettes for the past year, and smoked crystal meth and marijuana regularly. He denied injection drug use. Family history was pertinent only for diabetes mellitus in his father.

On physical exam, the patient was febrile (39.5 °C), tachycardic (112 bpm), normotensive (112/57 mmHg), had a normal respiratory rate (18 bpm) and oxygen saturation (97%). He was in no distress and fully oriented. He had bilateral cervical lymphadenopathy (∼1 cm, non-tender, mobile). His cardiopulmonary exam was within normal limits. His abdomen was unremarkable with no palpable hepatomegaly or splenomegaly. Head and neck, extremity, genital and neurologic exams were unremarkable. On skin evaluation, he had a violaceous papular-erosive rash in his neck.

On initial laboratory tests, the patient had leukopenia (3200 cells/mm3) with lymphopenia (500 cells/mm3), thrombocytopenia (81,000/mm3) and a normal hemoglobin level. A complete metabolic panel revealed mildly elevated creatinine (1.11 mg/dL), hypoalbuminemia (2.9 g/dL), hyponatremia (128 mmol/L), mild hypocalcemia (8 mg/dL) and mild transaminase elevation (AST 69 and normal ALT), normal bilirubin, and normal alkaline phosphatase levels. Inflammatory markers were significantly elevated (ESR 22 mm/hr; CRP 115 mg/dL). The urinalysis and a chest X-ray were unremarkable. COVID-19 PCR and influenza A/B PCR testing were negative. Blood cultures were collected. He received empiric vancomycin and cefepime in the emergency department and was admitted for further evaluation.

Further testing revealed significantly elevated ferritin (5379 ng/mL) and D-dimer (14.3 mcg/mL). Fibrinogen level, PT/INR, and aPTT were all within normal limits. He was also found to have elevated LDH (494 U/L) and triglyceride level (701 mg/dL). 4th generation HIV antigen/antibody testing and HIV RNA viral load were negative, rapid plasma reagin, Chlamydia/Gonorrhea nucleic-acid amplification from urine, viral hepatitis serologies (A, B, C), *M. tuberculosis* interferon-gamma release assay were negative/within normal limits. Further diagnostic testing for infectious etiologies were sent (Cytomegalovirus, Epstein-Barr virus, Brucella, Coxiella, Rickettsia, Coccidioidies serologies, Histoplasma antigen in urine, and mononucleosis heterophile antibodies). Extensive autoimmune antibody testing was also performed. A peripheral smear revealed marked thrombocytopenia, mild normochromic/normocytic anemia, and circulating plasma cells.

Computed tomography of chest, abdomen and pelvis revealed trace bilateral pleural effusions, slight increase in number of lymph nodes in mediastinum and bilateral hila, hepatomegaly, and trace pelvic free fluid.

Given concerns for HLH, the patient underwent a bone marrow biopsy and specific tests for this condition were sent, including a soluble interleukin-2 receptor (IL-2R) level and natural killer (NK) cell function assay. Given his persistent fever and concerns for FBT in setting of animal exposures, doxycycline was started empirically (100 mg po BID). A skin biopsy of the neck rash was also performed.

Following the initiation of doxycycline, the patient’s fever subsided, and his labs began to normalize, except for the transaminase levels. Chemistries, leukopenia, and thrombocytopenia all normalized, and his inflammatory markers significantly downtrended. The patient reported feeling much better in general.

**Fig. 1:**
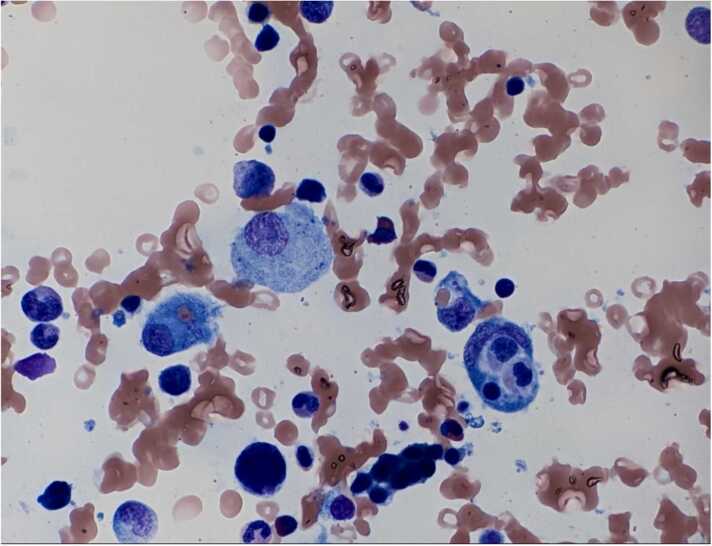
Bone marrow biopsy showing hemophagocytosis.

All of the blood tests for auto-immune and infectious etiologies returned negative, including *R. typhi* serologies. Testing for plasma cell dyscrasias including serum and urine protein electrophoresis, immunofixation, and free light chain assay in serum were unremarkable. The skin biopsy stains were negative for micro-organisms and revealed epidermal acantholysis suggestive of either Darier’s disease or Grover’s disease. The bone marrow studies revealed a hypocellular marrow with hemophagocytosis (see Fig. 1) and polytypic plasmacytosis. There was no evidence of malignancy, and stains for fungal/AFB organisms were negative.

Given his overall improvement by the end of a week into his hospitalization, the patient was discharged with the plan to complete a total of 7 days of doxycycline and to follow up in clinic, with repeat *R. typhi* serologies in 3 weeks. He was evaluated in clinic 4 weeks post discharge and reported feeling well. His repeat *R typhi* serologies were both highly positive IgM (>1:256) and IgG (>1:256), thereby confirming the diagnosis of flea-borne typhus. IL-2R levels came back elevated (10460 pg/mL), consistent with the diagnosis of HLH.

## Discussion

We have presented a case of a patient infected with *R. typhi* manifesting with HLH, an extremely uncommon but potentially fatal syndrome. It is well described that HLH syndrome can be triggered by infectious agents, including the rickettsial diseases scrub typhus, Mediterranean spotted fever and human monocytic ehrlichiosis [4], but there are few reports in the literature of *R. typhi*-associated HLH. A case report with review of the literature performed by Chandramohan et al. [5], revealed a total of 10 cases of HLH associated with FBT through 2022 [5], [6], [7], [8], [9], [10], [11], [12], [13]. Our case brings the total number of published cases to 11 with an overall mortality rate of 2/11 or 18% (see Table 1).

**Table 1:** Summary of Published Cases of HLH Associated with Flea-Borne Typhus.

Reference	Publication year and description	Age and Sex of patient	HLH diagnostic features	FBT serologies	Outcome
Miguelez et al.[6]	2003; Review of 32 cases of FBT in Tenerife (Spain) with one confirmed HLH (no further details provided)	NA	NA	NA	Improved
Walter et al.[7]	2012; Review of 32 cases of FBT in France with one confirmed HLH and 2 suspected (no further details provided)	NA	NA	NA	Improved
Loussiaef et al.[8]	2014; case report from Tunisia	52-year-old female	Hemophagocytosis on bone marrow Pancytopenia, fever, splenomegaly, hyperferritenemia, hypertriglyceridemia	Elevated *R. typhi* serologies (IgM and IgG) during acute phase	Improved
Wulff et al.[9] Iaria et al.[10]	2018; case report from Texas, USA	5-year-old female	Fever, anemia, thrombocytopenia, hyperferritenemia, hypertriglyceridemia, hypofibinogenemia,	Elevated *R. typhi* serologies (IgM and IgG) during acute phase with 4x increase in convalescent IgG titer	Improved
Poulos et al.[11]	2019; case report from Texas, USA	39-year-old male	Hemophagocytosis on bone marrow, Fever, thrombocytopenia, hypertriglyceridemia, hyperferritinemia	Elevated *R. typhi* serologies (IgM and IgG) during acute phase with > 4x increase in convalescent IgG	Improved
Leal-Lopez et al.[12]	2020; case report from Yucatan, Mexico	2-year-old male	Hemophagocytosis on bone marrow, fever, splenomegaly, pancytopenia, hypofibrinogenemia, hypertriglyceridemia	Neg serologies during acute phase; Positive *R. typhi* IgM and IgG during convalescent stage	Deceased
Jacqout et al.[13]	2022; case report from Lyon, France (patient returning from Reunion Island)	61-year-old male	Hemophagocytosis on bone marrow, Anemia, thrombocytopenia, hyperferritenemia, hypertriglyceridemia	> 4x increase in convalescent IgG for *R. typhi*	Improved
Chandramohan et al.[5]	2023; case report from Texas, USA	71-year-old female	Hemophagyctosis in liver, lymph nodes, spleen and bone marrow, fever, thrombocytopenia, splenomegaly, hypertriglyceridemia, hypofibrinogenemia	Positive *R. typhi* IgM during acute phase	Deceased
Present case	2024, this publication	32-year-old male	Hemophagocytosis on bone marrow, elevated IL-2R Fever, thrombocytopenia, hypertriglyceridemia, hyperferritinemia	Neg serologies during acute phase, Positive *R. typhi* IgM and IgG during convalescent stage	Improved

Diagnosing HLH can be challenging; the first set of diagnostic criteria was introduced in an international collaborative therapeutic study by the Histiocyte Society in 1994 (HLH-94) [14]. These were revised in a subsequent study (HLH-2004) and, to date, are still considered the gold standard for this diagnosis [15]. More recently, members of the HLH committee from the North American Consortium for Histiocytic Disorders (NACHO) compiled a review identifying pitfalls of the existing diagnostic criteria. The NACHO group review clarifies that HLH is a syndrome with a distinctive clinical pattern suggestive of an immune dysregulation, however, not all patients may benefit from immunosuppression. Only a subset of patients for whom the immune dysregulation is their core problem have “HLH disease,” and these patients may benefit from immunosuppressive therapy. However, those with an associated condition, such as an infectious process, leading to an HLH syndrome are classified as “HLH disease mimics” and would not benefit from immunosuppressive treatment [16].

The diagnosis of HLH is made either by finding heterozygosity of a verified HLH-associated mutation, along with clinical features associated with the syndrome or by fulfilling 5 of 9 clinical criteria. These include fever (>38.3 °C), splenomegaly, cytopenias (two of three cell lineages: Hb <9 mg/dL, platelets <100,000/mm3, absolute neutrophil count <1000/microL), hypertriglyceridemia (>265 mg/dL) and/or hypofibrinogenemia (<150 mg/dL), hemophagocytosis in bone marrow, spleen, lymph node, or liver, low-absent NK cell activity, ferritin > 500 ng/mL, elevated soluble CD25 (also known as soluble IL-2R), and elevated CXCL9 [15].

Based on these, our patient met the HLH diagnostic criteria given that he had fever, hypertriglyceridemia, hemophagocytosis on bone marrow biopsy, hyperferritenemia, and elevated IL-2R levels. He also had thrombocytopenia and leukopenia, although these did not reach cytopenia cut-off criteria. Our patient would be categorized as presenting with an “HLH disease mimic,” based on the definitions proposed by the aforementioned NACHO review [16].

With respect to the diagnosis of FBT, this should be suspected in patients with compatible clinical features and associated epidemiological risk factors [1]. In this case, we suspected FBT given the acute febrile illness associated with elevated transaminases, leukopenia, thrombocytopenia, exposure to rats, cats and opossums, and his residence in an endemic area for this disease. In Los Angeles County there has been a progressive increase in the number of flea-borne typhus cases reported in the past 5 years, with a record of 171 cases reported in 2022 (the year our patient presented), up from only 31 cases in 2010 [17]. Out of those 171 cases, there were 3 deaths. Of note, since 1993 there had been no deaths related to this disease reported in Los Angeles County.

As these bacteria cannot be easily cultured, serologic testing (IgM, IgG) continues to be the mainstay for diagnosis, but given delays in serologic test results, antibiotic treatment should be started empirically while awaiting confirmatory tests [18]. Patients rarely have positive antibodies during the first week of illness, and *R. typhi* serologic should be obtained both at the acute and convalescent stages of disease [1]. For our patient, we performed antibody serologic testing for *R. typhi* and initiated treatment with doxycycline, while waiting for the results. Initially, both IgG and IgM were negative (performed around day 7 after disease onset), but repeat testing after discharge (around week 3 from disease onset) revealed highly positive titers for IgM and IgG demonstrating seroconversion during the convalescent stage. Although FBT is associated with a low mortality, the HLH complication in this patient could have portended a more severe outcome. The empiric typhus treatment in this patient, despite negative initial serologies, was likely critical to a positive outcome for this patient.

## Conclusion

This case illustrates a very unusual, but aggressive presentation of infection with *Rickettsia typhi.* The natural history of HLH is said to be almost invariably fatal when left untreated. It is important to consider infectious agents as potential triggers for HLH syndrome, specifically *R. typhi* infection if the patient lives in an endemic area. Prompt empiric treatment for FBT should be considered, despite negative initial diagnostic results, with repeat serologies to be performed in the convalescent phase.

## Ethical approval

Case reports doesn’t require approval by ethics committee at our institution.

## Consent

Written informed consent was obtained from the patient for publication of this case report and accompanying images. A copy of the written consent is available for review by the Editor-in-Chief of this journal on request.

## Funding

No funding received for this manuscript.

## Declaration of Competing Interest

The authors declare that they have no known competing financial interests or personal relationships that could have appeared to influence the work reported in this paper.
